# Corrigendum

**DOI:** 10.1002/ccr3.6167

**Published:** 2022-07-25

In the article entitled “Friend or foe—The Pfizer‐BioNTech (BNT162b2) vaccination: A case report of reversible acute acalculous cholecystitis”,^1^ which was previously published in Volume 10 Issue 6 of Clinical Case Reports, a sentence was omitted in the Abstract section.

It has been updated as follows: “We described a rare case of vaccine‐induced acalculous cholecystitis (ACC). A 52‐year‐old woman developed ACC after 8 h of receiving a 3rd dose of the Pfizer‐BioNTech COVID‐19 vaccination. The symptoms subsided completely with conservative treatment for 12 days, and the ultrasound and laboratory findings went back to normal. The potential occurrence of septic constellation with acalculous cholecystitis after the 3rd dose of Pfizer‐BioNTech (BNT162b2) vaccination for protection against COVID‐19 is very rare and needs high index of suspicion and more investigation.”
